# Interspecific Cohabitation and Associated Zoonotic Risk in the Burrows of Niokolo‐Koba National Park, Senegal

**DOI:** 10.1002/ece3.74402

**Published:** 2026-10-28

**Authors:** Clara Russo, Yves Brostaux, Virginie Daelemans, Simon Lhoest, Johan Michaux, Mutien‐Marie Garigliany, Julie Bonnald, Fany Brotcorne, Justine Broers, Eric Heymans, Philippe Taminiaux, Ousmane Alioune Sylla, Mallé Gueye, Paul Moïse Diedhiou, Cédric Vermeulen

**Affiliations:** ^1^ Forest Is Life, Gembloux Agro‐Bio Tech University of Liège Gembloux Belgium; ^2^ Applied Statistics, Computer Science and Modeling Laboratory, Gembloux Agro‐Bio Tech University of Liège Gembloux Belgium; ^3^ Conservation Genetics Laboratory University of Liège Liège Belgium; ^4^ UMR 117 Animal Health, Territory, Risk and Environment University of Montpellier, CIRAD, INRAE Montpellier France; ^5^ Laboratory of Veterinary Pathology, FARAH ‐ CIRM – INDEEP University of Liège Liège Belgium; ^6^ Paris Zoological Park National Museum of Natural History Paris France; ^7^ SPHERES Research Unit, Behaviour Biology Unit, Department of Biology, Ecology, and Evolution, Faculty of Sciences University of Liège Liège Belgium; ^8^ Nioko Lodge Tambacounda Senegal; ^9^ Department of HydroSciences and Environment Iba Der Thiam University of Thiès Thiès Senegal; ^10^ Niokolo‐Koba National Park National Parks Directorate Tambacounda Senegal

**Keywords:** camera traps, coexistence, feces, one health, ticks, zoonoses

## Abstract

In arid and semi‐arid ecosystems, burrows serve as vital sanctuaries providing wildlife shelter, nourishment, and thermal buffering. These benefits attract multiple species into confined spaces, potentially facilitating interspecific pathogen transmission. This study provides a comprehensive assessment of burrow use in Senegal's Niokolo‐Koba National Park, quantifying interspecific cohabitation, identifying influential characteristics, and documenting the potential for zoonotic pathogen transmission. Data were collected during two dry‐season campaigns, which included semi‐opportunistic searches of burrows for camera‐trap monitoring and collection of fecal and tick samples. Cameras detected entering species while samples targeted bacteria with zoonotic potential. Across 1519 camera‐trap days, 32 species were detected using burrows, generating 172 interspecific cohabitations. Each cohabitation represented a species pair entering at least one common entrance during monitoring. An average of 6 different cohabitations per entrance over 20 days was recorded, with a maximum of 5 species visiting the same entrance within 24 h. Occupation occurred in rapid succession rather than simultaneously. Twenty significant preferential cohabitations were identified, ranging from ecological partnerships to predator–prey co‐occurrences. Based on the characteristics recorded for 105 monitored entrances, larger entrance diameter, higher woody cover, and location in valleys were associated with higher cohabitation rates. Pathogen screening detected potential zoonotic bacteria, including *Treponema*. Ticks were found in half of sampled burrows, with *Francisella* consistently detected. Results underline the dual ecological and epidemiological roles of burrows, emphasizing the need to integrate One Health considerations into burrow conservation.

## Graphical abstract

Burrows concentrate multiple species in confined spaces, potentially facilitating pathogen transmission. Camera traps at 105 burrow entrances in Niokolo‐Koba National Park documented 32 species generating 172 interspecific cohabitations. Potential zoonotic bacteria (*Treponema*, *Francisella*) were detected, and ticks were found in half of sampled burrows, highlighting the need for One Health conservation strategies.

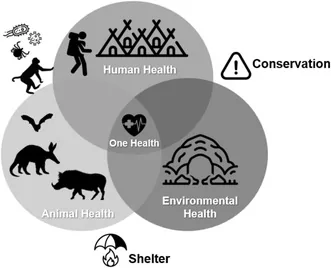

## 1 Introduction

Arid and semi‐arid ecosystems are exposed to severe abiotic constraints such as extreme temperatures and limited water availability, which are further intensified by climate change (Timis‐Gansac et al. 2025). According to the United Nations Convention to Combat Desertification (2024), 77.6% of Earth's land surface has become drier over the past three decades, mainly due to climate‐driven aridification. This process has led to a gradual loss of biodiversity, with animal species being particularly vulnerable. Mammals struggle to maintain their thermal balance in response to more frequent and intense heatwaves (Fuller et al. 2021), while mass die‐offs have been reported among desert birds in California (Iknayan and Beissinger 2018).

Burrows are among the most critical structures for survival in extreme environments (Whittington‐Jones et al. 2011). They are underground spaces dug by different animal species, with complexity ranging from simple linear tunnels to branched systems with multiple chambers and multiple entrances (Meadows PS and Meadows A 1992). Primary diggers, defined as species that excavate their own burrows, are predominantly mammals. Species that frequently use burrows excavated by other animals are referred to as commensals. These include secondary modifiers, which alter the structure of existing burrows, and non‐modifying species, which occupy burrows without changing their structure. Commensals can be classified as obligate, when they are strongly associated with a particular burrow type, or non‐obligate, when they may also use other types of shelter (Kinlaw 1999). Using existing burrows allows them to avoid the energetic cost of digging while benefiting from the burrow's advantages, such as temperature regulation, moisture conservation, protection from fire, and better sound transmission that support communication (Kinlaw 1999; Whittington‐Jones et al. 2011).

However, the resulting interspecific cohabitation creates significant health risks (Lehmann et al. 2020). Because burrows are places of frequent and diverse animal interactions, they must be recognized as potential zoonotic hotspots, in line with the “One Health” approach (Vermeulen et al. 2025), which emphasizes the interdependence of human, animal, and environmental health (World Health Organization 2022a). The World Health Organization has reported a 63% increase in zoonotic diseases in Africa over the last decade (World Health Organization 2022b). Several studies conducted in Senegal's Niokolo‐Koba National Park (NKNP) help to understand the mechanisms that may lead to interspecific transmission within burrows.

Animal waste, such as feces, urine, and secretions found in burrow chambers, can contain live pathogens. Zoonotic gastrointestinal parasites like *Strongyloides stercoralis* and *Trichuris* sp. have been identified in the feces of Guinea baboons ( *Papio Papio* ) in the NKNP (N'da et al. 2022). A previous study also showed that Guinea baboons use burrows in the park (Vermeulen et al. 2025), suggesting that zoonotic parasites may be introduced into these structures through their feces.

Burrow‐dwelling arthropods also increase disease transmission risks. Ticks transmit more pathogen species to humans, livestock, and wildlife than any other blood‐sucking arthropod (Durden 2006). *Ornithodoros sonrai* , a vector of *Borrelia* spp., the causative agent of tick‐borne relapsing fever, has been found in small mammal burrows near human settlements in several Senegalese villages (Ndiaye et al. 2023).

Close social interactions inside burrows, such as grooming, fighting, or mating, can facilitate the direct transmission of pathogens both within and between species (Kappeler et al. 2015). Mediannikov et al. (2020) reported a treponematosis outbreak in green monkeys ( *Chlorocebus sabaeus* ) in the NKNP, followed by the detection of a closely related clone in Guinea baboons ( *Papio papio* ), suggesting potential interspecific transmission. The infection was caused by *Treponema pallidum* subsp. *pertenue*, a typically non‐venereal pathogen that appeared to be sexually transmitted during this outbreak. This example illustrates how close‐contact behaviors can facilitate pathogen transmission between wildlife species.

The NKNP provides a relevant setting for studying interspecific cohabitation and its associated zoonotic risks. It hosts rich biodiversity, including over 70 mammal species and 329 bird species, along with many reptiles, amphibians, and invertebrates (UNESCO Centre du patrimoine mondial 2025). The aardvark ( *Orycteropus afer* ) is the main burrow‐digging species in the park. This nocturnal mammal is highly adapted for digging, with strong hind limbs and a tail that helps move soil (Melton 1976). It constructs several types of burrows, including shallow foraging holes, temporary shelters, and complex permanent dwellings (Smithers 1971). In Africa, aardvark burrows are known to support a high diversity of commensal species, with 27 vertebrate species recorded over one year (Whittington‐Jones et al. 2011) and up to five species visiting a single burrow within 24 h (Haussmann et al. 2023). Additionally, the proximity of nearby villages to wildlife habitat, combined with poaching and tourism activities, contributes to increased human‐wildlife interactions. Guinea baboons ( *Papio papio* ) and green monkeys ( *Chlorocebus sabaeus* ), often visit tourist camps, creating opportunities for direct contact with humans (Mediannikov et al. 2020).

This study aims to (i) quantify the extent of interspecific cohabitation within burrows monitored during two field surveys and describe species associations; (ii) identify which environmental and structural characteristics of burrows influence interspecific cohabitation; and (iii) document the zoonotic risks linked to burrow use by analyzing fecal and tick samples collected at their entrances.

## 2 Methods

### 2.1 Study Area

Niokolo‐Koba National Park (NKNP) is in southeastern Senegal, covering 913,000 ha between 12°30′–13°20′ N and 13°42′–18°30′ W. It is crossed by the Gambia River and its main tributaries (UNESCO Centre du patrimoine mondial 2025). The park was designated a UNESCO World Heritage Site in 1981 and listed as endangered in 2007 due to poaching, livestock encroachment, and the Sambangalou dam project. Following successful conservation measures, it was delisted in 2024 (Houéhounha et al. 2025). The climate is tropical savanna (Aw) according to the Köppen–Geiger classification. Topography is generally flat, with elevations ranging from 16 to 311 m, reaching a maximum at Mount Assirik (Camara et al. 2023).

The research was conducted around Niokolodge, a tourist camp located in the Tambacounda Region (Figure 1). The area features diverse soil types including lithosols on cuirasses, tropical ferruginous and hydromorphic soils (Maignien 1965). Savannas dominate the landscape, ranging from shrub savannas characterized by a continuous herbaceous layer with sparse shrubs, to woody savannas (5%–25% woody cover) and savanna woodlands (25%–50% woody cover). Their common characteristics include a continuous herbaceous layer, the predominance of Poaceae and a high density of Combretaceae (Mbow et al. 2003). Open forests (50%–75% tree cover) composed of Combretaceae, Fabaceae, Meliaceae, Ochnaceae, and Phyllanthaceae, along with localized bamboo formations ( *Oxytenanthera abyssinica* ), also occur (Arbonnier 2002).

**FIGURE 1:**
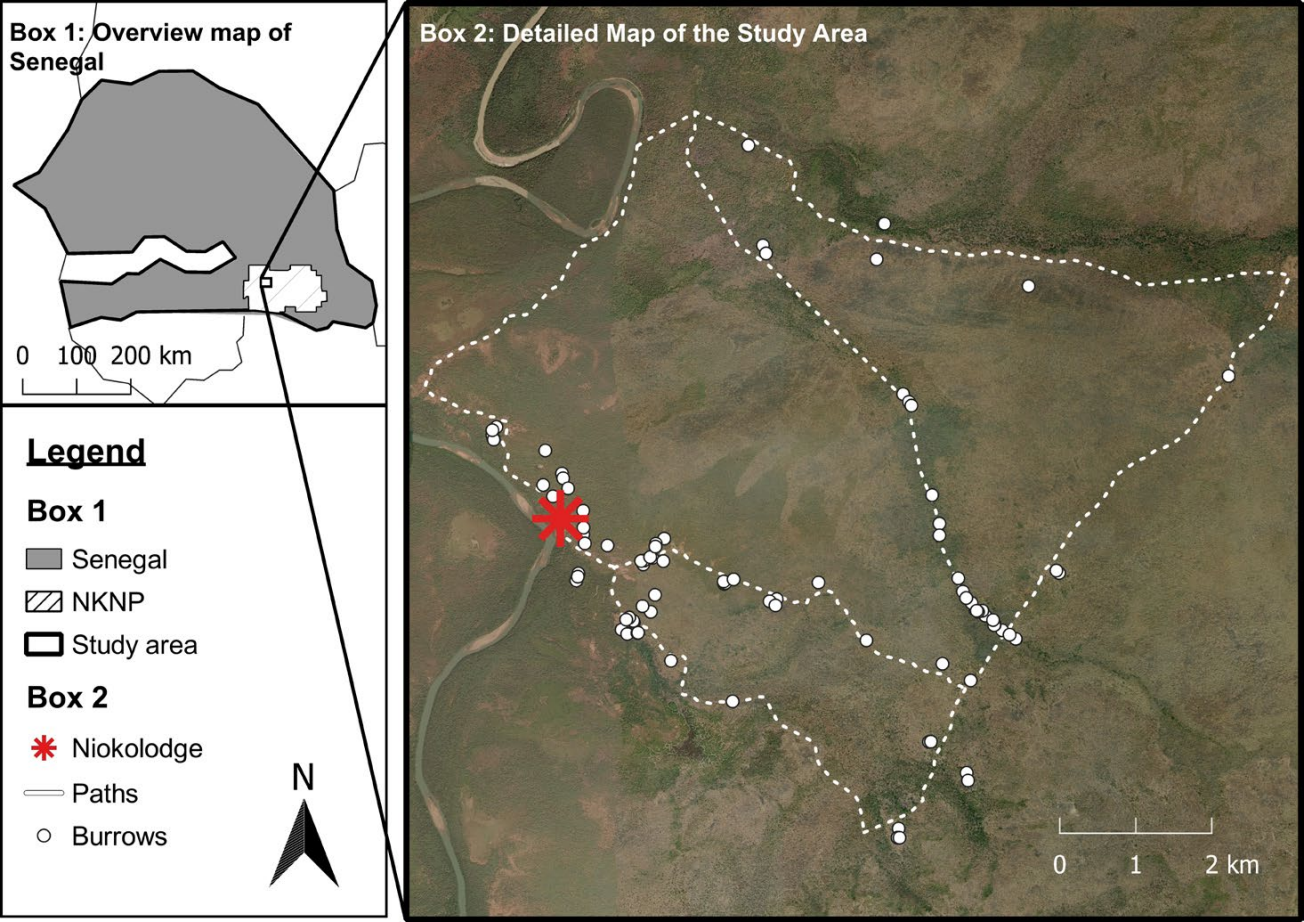
Overview map of Senegal showing Niokolo‐Koba National Park and the study area location (Box 1) followed by a satellite map of the study area (Box 2) indicating 88 selected burrows (white circles) along surveyed routes (dashed lines) with Niokolodge (ecolodge) used as a geographical reference (sources of spatial data: ESRI Satellite basemap).

### 2.2 Data Collection

Data collection was conducted during two field campaigns in the dry season (27 Feb‐1 May 2023 and 30 Jan‐10 Apr 2025). Both campaigns employed camera traps and fecal sample collection. Additionally, soil samples and tick samples were collected during the first and second campaigns, respectively.

#### 2.2.1 Search and Characterization of Burrows

A semi‐opportunistic search was conducted. During each field campaign, all vehicle‐accessible tracks surrounding the camp were explored, totaling approximately 53 km. In areas with sufficient visibility, foot surveys were conducted up to 500 m from the road. Complex burrows (≥ 2 entrances) and simple burrows (1 entrance, > 30 cm diameter) were selected. Selection criteria included visible recent animal traces (tracks, droppings, fresh diggings; Suuri et al. 2021) and reduced or absent leaf litter at the entrance.

For each selected burrow, the geographic coordinates were recorded using a Garmin 66 s GPS device. Several site‐specific characteristics were noted, including geomorphology (i.e., plateau, slope, ridge or valley), vegetation formation (i.e., shrub savanna, woody savanna, savanna woodland, open forest or bamboo formation), woody cover above the entrance (0%–100%), soil type (based on texture analysis of 25 mL samples; Appendix S1), and burn stage (i.e., recent: abundant black traces and minimal regrowth; intermediate: some charcoal and vegetation regrowth; old: tall grasses and no visible charcoal).

Specific entrance‐scale characteristics were recorded, including minimal and maximal diameters, and the presence of animal or insect traces. Occupancy level was estimated using a three‐level scale: heavily frequented (smoothed walls > 70% and < 10 leaves), moderately frequented (smoothed walls 50%–70% and 10–20 leaves), and not/lightly frequented (smoothed walls < 50% and > 20 leaves). For complex burrows, only entrances meeting the selection criteria were retained for data collection. Since underground connections between entrances could not be systematically confirmed, the entrance was considered the independent sampling unit. The field data sheet and photographs illustrating occupancy levels are available in Appendices S2 and S3.

#### 2.2.2 Installation of Camera Traps

Burrow entrances were monitored using Bushnell Core DS‐4 K cameras for 10 or 20 days across the two study periods. An initial 10‐day monitoring period represented a compromise between available field time, the limited number of 46 cameras, and the need to monitor enough burrows for robust statistical analyses. However, species accumulation curves from the first campaign showed that new species were still being detected until the final days of monitoring at most burrow entrances (Appendix S4). The monitoring duration was therefore extended to 20 days for most burrows during the second campaign. Cameras were positioned on trees, shrubs, or wooden stakes near burrow entrances. When possible, they were placed in shade to limit heat exposure and false triggers from the passive infrared motion sensor (PIR) (Welbourne et al. 2016). Simple burrows were monitored with a single camera, while complex burrows required up to three cameras. Cameras were configured in video mode with medium resolution to avoid saturating 64 GB SD cards. Fast motion setting was activated for rapid detection (Bushnell 2021). Videos lasted 15 s with 0.5 s trigger delay and operated continuously 24/7 (Fonteyn et al. 2025). The PIR sensor was set to “High” for optimal detection above 40°C (Bushnell 2021).

#### 2.2.3 Collection of Samples

Feces were collected at the time of camera installation and checks. The area around burrow entrances was examined, and droppings were collected using a spatula and surgical gloves to avoid contamination. Samples were stored in 25 mL tubes filled with 98% ethanol (Taberlet et al. 2018) and stored at −20°C. Each tube was labeled with the suspected producing species, the collection date, and the entrance identification.

Ticks were collected at 10 burrows monitored by camera traps. The number of sampled burrows was limited by budgetary constraints associated with laboratory analyses. For each vegetation type, two burrows with an occupancy level ≤ 2 (i.e., moderate or heavy use) were selected during the installation of camera traps. Two methods were used for tick collection.

The first method (hereafter the “scraping method”) used a trowel with a 1 m wooden handle to scrape the four walls of the burrow entrance (three passes per wall). Soil was spread on white paper and exposed to sunlight to detect ticks, which were collected with a tweezer (Jori et al. 2013). Ticks were preserved in 2 mL tubes containing 98% ethanol (Taberlet et al. 2018) and stored at −20°C. Ticks from the same burrow entrance were pooled in the same tubes. Each container was labeled with the date, burrow entrance identification, and the number of individuals inside. The second method (hereafter the “heat‐attraction method”) followed the protocol described by Bansal et al. (2019), which was applied to kiwi burrows. An 800 mL hot water bottle (36 × 19 cm) in a fleece cover was inserted into burrows using 90 cm forceps. The bottle remained in the burrow for approximately 1.5 h (time required to cool to 38°C at an ambient temperature of 28°C). After removal, it was stored in a cellophane bag at 4°C for 2 h before tick collection and preservation as described above.

### 2.3 Data Analysis

#### 2.3.1 Species Detection and Cohabitation Analysis

Camera trap videos were manually reviewed to exclude false triggers and analyzed with *Timelapse2* software. For each sequence, several parameters were recorded: observed species, number of individuals, detection time (day or night), and level of interest in the burrow entrance (categorized as no interest, interest, or penetration; Vermeulen et al. 2025). Interest was defined as any behavior directed toward the burrow entrance (e.g., sniffing, observation, urine marking), while penetration corresponded to partial or complete crossing of the entrance by at least one individual. Two species complexes were defined due to uncertain identifications: the “mice complex” (including Muridae and unidentified families) and the “lizards complex” (including Agamidae, Scincidae, and Gekkonidae). Detections were aggregated into independent events. For each species and burrow entrance, detections separated by less than 30 min were grouped within the same event. Within each event, one detection was retained, prioritizing the highest level of interest (penetration > interest > no interest).

Only entrances with at least one recorded penetration were retained. Cohabitation is defined here as the penetration of two species into at least one common entrance during the monitoring period (Vermeulen et al. 2025). For each entrance, all pairs of species that had penetrated were examined. Thus, an entrance used by *n* species corresponded to *n*(*n* − 1)/2 distinct species pairs, each considered a cohabitation. For each species pair, we calculated spatial distribution (percentage of shared entrances) and temporal frequency (percentage of days with same‐day use). In addition, each species pair was tested using Fisher's exact test to determine whether cohabitation was preferential, avoidant, or random. Cohabitation was considered significantly preferential when observed co‐occurrence frequencies exceeded those expected by chance, significantly avoidant when lower, and random when not significantly different from chance expectations.

To account for differences in monitoring duration among entrances, two generalized linear models (GLMs) were used. First, the total number of cohabitations observed per entrance was modeled using a negative binomial distribution, with log‐transformed monitoring duration included as an offset and no ecological fixed effect. This model estimated the mean number of cohabitations per entrance over 20 days. Second, logistic regression modeled the presence or absence of cohabitation according to monitoring duration. It estimated the probability of observing at least one cohabitation per burrow entrance over 20 days. These models were developed given that most burrows were still in the linear phase of the accumulation curve after 10 days during the first campaign.

To assess whether individual burrow characteristics influenced interspecific cohabitation, each characteristic was tested separately using a univariate negative binomial model. The number of cohabitations per entrance was the response variable, with the logarithm of monitoring duration included as an offset. Models were evaluated based on AIC and likelihood‐ratio test (LRT) significance (*p* < 0.05).

All statistical analyses were conducted in RStudio version 4.5.0, notably using the packages, dplyr and MASS.

#### 2.3.2 Genetic Analysis of Fecal and Tick Samples

DNA was extracted from fecal and tick samples using the *QIAamp DNA Mini Kit* (Qiagen). Several fragments from each fecal sample were homogenized together in InhibitEX solution before extraction to reduce PCR inhibitors. Ticks from the same tube were crushed together using a plastic pestle and incubated overnight at 56°C with ATL buffer and proteinase K before extraction. The pestle was decontaminated between samples using three successive bleach baths, followed by rinsing and drying.

PCR amplification targeted: (1) the V3–V4 region of the bacterial 16S rRNA gene for fecal and tick samples (Klindworth et al. 2013); (2) the mammalian mitochondrial 12S marker for fecal samples collected in 2025 (Riaz et al. 2011); and (3) the CO1 marker for tick samples (Hebert et al. 2003). Amplicons were purified with AMPure XP magnetic beads, and sample‐specific indices with Illumina adapters were added via a second PCR. DNA concentrations were quantified using the *PicoGreen dsDNA Assay Kit*, normalized to equimolar concentrations, and pooled. Libraries were sequenced on an Illumina NovaSeq platform (1 million reads for 12S; 2 million reads for CO1 and 16S).

Low‐quality sequences were filtered, and taxonomic assignments were performed by comparing sequences against reference databases: NCBI for mammalian 12S, BOLD for CO1, and RDP Classifier for bacterial 16S (Wang et al. 2007). To retain only reliable identifications, sequences from the 12S and CO1 markers were filtered to include only those with a match distance > 80% and overall match > 90% with reference sequences. For bacterial 16S data, reads with counts < 1000 were excluded, and only taxonomic assignments with bootstrap support ≥ 0.8 were retained for downstream results.

## 3 Results

A total of 88 burrows were selected for the study, representing 105 entrances (54 in 2023 and 51 in 2025). Camera monitoring lasted 10 days (occasionally up to 13) for 59 entrances and 20 days for 46 entrances. This represents a total of 1519 camera‐days. One complex burrow monitored in 2023 remained active in 2025. Of its four entrances recorded in 2023, three remained open and were monitored again in 2025. These entrances were considered independent units from year to year. An abandoned termite mound with an opening larger than 30 cm was also included in the survey.

### 3.1 Interspecific Cohabitation Extent and Species Associations

The extent of interspecific cohabitation was assessed at three hierarchical levels: the study area, the burrow entrance, and the interaction scales. As a reminder, each cohabitation refers to a species pair, with all possible pairs examined when > 2 species occupied the same burrow entrance (occurring in > 75% of cohabited entrances; Appendix S5).

At the scale of the study area, extent was estimated as the number of cohabitations observed across all monitored burrow entrances and the probability that burrow entrance exhibits at least one cohabitation. In total, 172 different cohabitations were recorded, 20 were significantly preferential according to Fisher's exact test. The likelihood that at least two different species enter the same burrow entrance within 20 days is 93%, calculated from the entire dataset and standardized to 20 days (Appendix S6). These results stem from the detection of 32 species entering burrows, out of a total of 60 species detected across the study area (Appendix S7).

All species pairs showing non‐random cohabitation are presented in Table 1. They all reflect significant preferential cohabitation, suggesting that these species neither avoid each other nor co‐occur randomly. The strongest associations were indicated by infinite odds ratios (ORs), with 12 species pairs always occurring together in the same burrow entrance. Other associations exhibited odds ratios ranging from 4.6 for the *Chiroptera* × Mice complex to 55.5 for the spotted hyena ( *Crocuta crocuta* ) × Gambian mongoose ( *Mungos gambianus* ). An odds ratio of 4.6 suggests that the likelihood of encountering a mouse in a burrow is 4.6 times higher when a bat is present than when it is absent.

**TABLE 1:** Non‐random interspecific cohabitations detected using Fisher's exact test.

Pair of species	OR
*Camaroptera brachyura × Cricetomys gambianus*	Infinite
*Camaroptera brachyura × Francolinus bicalcaratus*	Infinite
*Camaroptera brachyura × Mungos mungo*	18.7
*Chiroptera ×* Mice complex	4.6
*Chiroptera ×* Lizards complex	Infinite
*Cossypha albicapilla × Turdoides reinwardtii*	Infinite
*Cricetomys gambianus × Francolinus bicalcaratus*	Infinite
*Crocuta crocuta × Hystrix cristata*	Infinite
*Crocuta crocuta × Mungos gambianus*	55.5
*Genetta genetta × Mungos gambianus*	Infinite
*Erythrocebus patas × Eremomela pusilla*	Infinite
*Genetta genetta × Python sebae*	Infinite
*Heliosciurus gambianus × Mungos mungo*	16.1
*Herpestes ichneumon ×* Mice complex	4.524
*Herpestes ichneumon ×* Lizards complex	6.7
*Hystrix cristata × Mungos gambianus*	Infinite
*Malaconotus blanchoti × Tchagra senegalus*	55.5
Mice complex *×* Lizards complex	8.7
*Mungos gambianus × Python sebae*	Infinite
*Mungos gambianus ×* Lizards complex	Infinite

*Note:* Each row shows a species pair, with odds ratio (OR) indicating the strength of association between the two species. OR > 1 indicates that species co‐occur more frequently than expected by chance (preference), and infinite values indicate perfect association where species are never found without each other.

At the burrow scale, extent was estimated by the average number of cohabitations per entrance over 20 days (standardized from the entire dataset) and the maximum species richness within 24 h. On average, 6 different cohabitations occurred over 20 days, with a maximum of 5 species detected in 24 h.

At the interaction scale, extent was defined by the spatial distribution across burrow entrances and the temporal frequency within these burrow entrances. The cohabitation matrix (Figure 2) presents two values for each species pair. The cells above the diagonal indicate the proportion of burrow entrances shared by both species during the study period (spatial distribution), while the cells below the diagonal show the percentage of recording days when both species used those entrances on the same day (temporal frequency).

**FIGURE 2:**
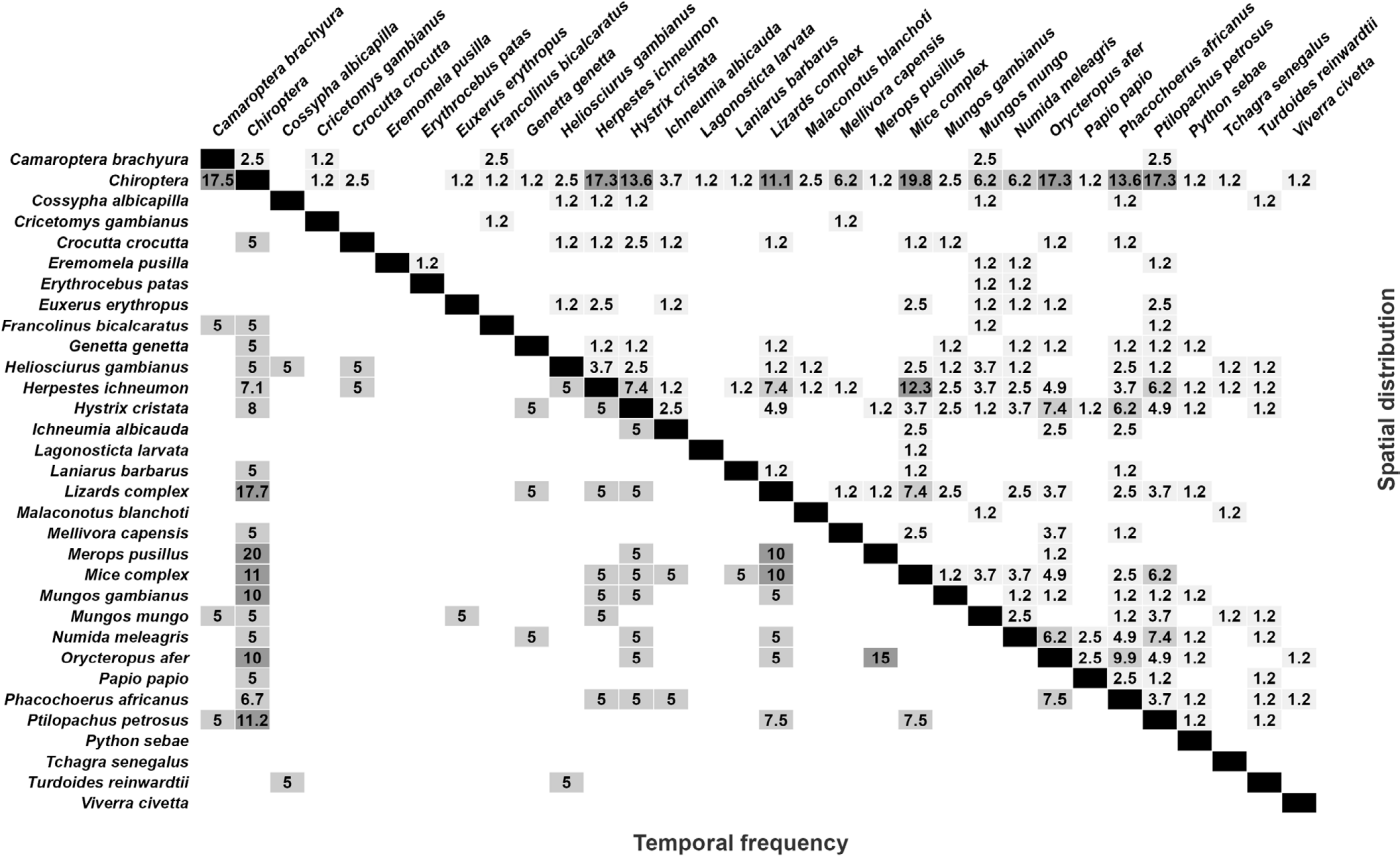
Cohabitation matrix. Each cell in the upper triangle represents the percentage of burrow entrances used by both species during the study period (spatial distribution). Each cell in the lower triangle displays the percentage of recording days when both species used entrances on the same day (temporal frequency). Darker shading indicates higher values (legend: 1%–5%, 5%–10%, 10%–25%). The matrix is based on non‐standardized data, combining burrows with monitoring durations from 10 to 20.

In terms of spatial distribution, some species occupy many burrows and exhibit broad overlap with other species. Bats (*Chiroptera*) were detected in 60% of entrances, sharing burrows with 27 of the 31 other species. High spatial values were observed with the Mice complex (19% of entrances), Egyptian mongoose ( *Herpestes ichneumon* ) (17%), aardvark ( *Orycteropus afer* ) (17%), and stone partridge ( *Ptilopachus petrosus* ) (17%). Other species had more restricted spatial distributions with focused associations. The Egyptian mongoose ( *Herpestes ichneumon* ) was present in 27% of entrances and mainly co‐occurred with the Mice complex (12%), the Lizards complex (7%), and crested porcupine ( *Hystrix cristata* ) (7%). The aardvark ( *Orycteropus afer* ) occurred in 23% of entrances and shared entrances with common warthog ( *Phacochoerus africanus* ) in 9% of cases.

In terms of temporal frequency, several species pairs shared the same burrows, but only some were recorded together over a high proportion of days. Notable examples included little bee‐eater ( *Merops pusillus* ) and bats (*Chiroptera*) (20% of recording days), bats and the Lizards complex (17%), and gray‐backed camaroptera ( *Camaroptera brachyura* ) and bats (17%).

### 3.2 Influence of Burrow Characteristics on Interspecific Cohabitation

Several individual burrow characteristics were significantly associated with the cohabitation rates (Table 2). Average entrance diameter constituted the best‐fitting predictor of cohabitation rates (AIC = 382.0, *p* = 0.0119), followed by the number of open entrances (AIC = 382.1, *p* = 0.0134), geomorphology (AIC = 382.9, *p* = 0.0197), woody cover (AIC = 383.5, *p* = 0.0303), and the total number of entrances (AIC = 384.2, *p* = 0.0481).

**TABLE 2:** Results of univariate models for cohabitation rates.

Characteristics	AIC	p(LRT)	Signif.
Average entrance diameter	382.0	0.0119	*
Open entrances	382.1	0.0134	*
Geomorphology	382.9	0.0134	*
Woody cover	383.5	0.0303	*
Total entrances	384.2	0.0481	*
Vegetation type	386.5	0.0687	ns
Insect activity	386.4	0.2016	ns
Estimated occupancy level	387.2	0.3488	ns
Animal tracks	387.4	0.4031	ns
Burned soil	388.1	0.9941	ns
Soil type	390.7	0.3533	ns

*Note:* Lower AIC and p(LRT) values indicate better model fit and stronger evidence that the burrow characteristic explains variation in cohabitation rates. * = significant effect.

Abbreviation: ns, not significant effect.

Specifically, cohabitation rates were significantly higher in valleys than on plateaus (estimate = 1.189, SE = 0.433, Tukey‐adjusted *p* = 0.031). For continuous variables, the number of open entrances (estimate = −0.386, SE = 0.148) and the total number of entrances (estimate = −0.143, SE = 0.069) were negatively associated with cohabitation rates, whereas woody cover (estimate = 0.021, SE = 0.009) and average entrance diameter (estimate = 0.052, SE = 0.020) were positively associated with cohabitation rates.

### 3.3 Taxa Detected in Biological Samples

#### 3.3.1 Fecal Samples

A total of 13 samples were collected, with eight in 2023 and five in 2025. Fecal origin was determined by 12S rRNA gene sequencing (visual identification for 2023 samples), and bacterial diversity by 16S rRNA gene sequencing. Eight bacterial families with zoonotic potential were identified, exhibiting varying distributions among host species (Table 3). Details of the fecal sampling (collection date, producing species, and distance to the entrance) and the complete list of detected bacterial families are available in Appendices S8 and S9.

**TABLE 3:** Presence of bacterial families with zoonotic potential in host fecal samples.

Bacterial family	Porcupine (*n* = 4)	Warthog (*n* = 3)	Baboon (*n* = 1)	Antelope (*n* = 1)	Unidentified (*n* = 4)
Bacillaceae	++	++	−	+	++
Campylobacteraceae	−	−	−	−	++
Clostridiaceae	++	++	−	−	+
Enterobacteriaceae	++	−	−	−	++
Mycoplasmoidaceae	+	−	−	−	−
Peptostreptococcaceae	++++	++	−	−	++
Spirochaetaceae	++	++	+	−	−
Streptococcaceae	++	−	−	−	+

*Note:* − = not detected, + = detected in 1 sample, ++ = 2 samples, ++++ = 4 samples.

#### 3.3.2 Tick Samples

Tick samples were collected from each of the 10 selected burrows. However, only 5 of these burrows had ticks present. A total of 101 individual ticks were collected, comprising 85 ticks collected with the scraping method and 16 ticks collected with the heat‐attraction method (Appendix S10). CO1 sequencing provided two complementary types of information: identification of the tick species (non‐chordate markers) and identification of the vertebrate host it fed on (chordate markers).

Tick identification revealed a clear dominance of the genus *Haemaphysalis* (90%–98% of filtered sequences per sample), with the closest matching species being *H. concinna* and *H. hoodi* . However, match rates were modest (90%–94%), likely due to incomplete reference database for African ticks, preventing confident species‐level identification. The genus‐level assignment corroborates previous findings, as *Haemaphysalis* has been reported across all three administrative regions of the park (Sylla et al. 2018). Although most species in this genus are not primarily endophilic, this behavior has been documented for *H. hispanica* in rabbit burrows (Estrada‐Peña et al. 1992). Regarding blood meal identification, DNA from the common warthog ( *Phacochoerus africanus* ) was detected. The high abundance of these sequences (8%–16% of filtered reads within a sample) and the high similarity percentages with the database (97%–100%) confirm the reliability of identifying *P. africanus* as the last blood meal of the analyzed ticks.

The 16S rRNA gene sequencing was used to profile the bacterial communities. At least one bacterial family with zoonotic potential was detected in each tick‐infested burrow. In total, six of such families were identified, with varying distributions across burrows (Table 4). The complete list of detected bacterial families is available in Appendix S11.

**TABLE 4:** Distribution of bacterial families with zoonotic potential across tick‐infested burrows.

Bacterial family	g_66	g_67	g_69	g_80	g_85	Total burrows
Bacillaceae	−	+	+	−	−	2/5
Corynebacteriaceae	+	+	−	−	+	3/5
Francisellaceae	+	+	+	+	+	5/5
Moraxellaceae	+	+	+	−	−	3/5
Mycobacteriaceae	+	−	+	−	−	2/5
Staphylococcaceae	+	+	+	−	−	3/5

*Note:* g_66–g_85 = burrow IDs; − = not detected, + = detected.

## 4 Discussion

### 4.1 Interspecific Cohabitation Extent and Species Associations

A total of 32 species were observed entering at least one burrow, resulting in 172 cohabitations over 1519 camera‐days. This exceeds the 27 species recorded by Whittington‐Jones et al. (2011) during a year‐long study in South Africa. Both studies confirm the use of aardvark burrows by Carnivores, Suiformes, Lagomorphs, Passeriformes, Squamates, and Rodents, while the current survey also includes Primates and Chiroptera. In contrast to their findings, no amphibians were recorded in our dataset, despite burrow humidity theoretically favoring their presence. These differences are likely explained by the methods used for data collection. Whittington‐Jones et al. relied on direct observations and Sherman traps. In contrast, our protocol used passive camera traps with PIR sensors, which are effective at detecting warm‐blooded animals but less effective at detecting cold‐blooded species (Hobbs and Brehme 2017). A combined approach incorporating both active and passive methods could provide a more comprehensive assessment of burrow users.

Up to five species were recorded entering the same burrow within 24 h, consistent with the findings of Haussmann et al. (2023), who reported the same number in aardvark burrows in South Africa despite a lower sampling effort of 44 camera‐days. This suggests a possible upper limit to daily burrow use across species, likely due to temporal partitioning in burrow use observed in both studies. Indeed, observations indicate that species tend to follow one another in burrows rather than co‐occupying them simultaneously. Of the 172 interspecific cohabitations recorded, 114 pairs of species were never observed using the same entrance on the same day. However, these successions are not spaced in time: the probability of observing at least two species using the same entrance within 20 days is estimated at 93%, with an average of 6 cohabitations recorded over that time.

Among the 20 significant preferential cohabitations identified, several ecological patterns emerge. Some of these co‐occurrences reflect common ecological needs. This interpretation can be applied to the associations between the gray‐backed camaroptera ( *Camaroptera brachyura* ) and the banded mongoose ( *Mungos mungo* ), and between the patas monkey ( *Erythrocebus patas* ) and the Senegal eremomela ( *Eremomela pusilla* ). Mongooses and certain primates were recorded scratching the burrow walls, likely in search of insects or larvae (Kingdon 2015), which could explain their co‐occurrence with insectivorous birds. Some cohabitations rather reflect predator–prey dynamics. For instance, *the* Egyptian mongoose ( *Herpestes ichneumon* ) cohabits with mice and lizards, two key prey taxa in its diet (Kingdon 2015). Another example involves the spotted hyena (*Crocutta crocutta*), which was recorded inserting only their forelimbs into burrows and remaining motionless for several minutes, presumably adopting a sit‐and‐wait predation strategy. These findings suggest that beyond strict predatory interactions, a certain degree of tolerance may exist, particularly under climatic stress. Prey species must balance the benefits and risks of using burrows as sites for foraging and shelter.

Other cohabitations, although present in a large proportion of burrows, were not significantly preferential. This may seem counterintuitive, but it can be explained by the fact that some taxa, such as bats, are commonly present in burrows (out of the 1260 penetration events recorded, 655 were attributed to bats; Appendix S7). Their high individual occurrence increases the probability of cohabiting with other species. These cohabitations primarily reflect the general tendency of these species to occupy burrows, rather than any specific ecological affinity with other species. In contrast, the random nature of some associations was to be expected, as with the aardvark. Many species occupy aardvark burrows when they seem abandoned (Kinlaw 1999). However, aardvarks rarely dug new burrows, instead moving into one of the many old ones (Taylor and Skinner 2003), potentially leading to cohabitation upon their return. Moreover, the average duration of continuous occupation by an aardvark is relatively short (4 to 8 days) (Taylor and Skinner 2003), which limits temporal overlap with other species and explains the low frequency of shared use of entrances.

Some co‐occurrences may not have been detected in this study. Although the monitoring period was extended from 10 to 20 days between the first and second field campaigns, new species were still recorded at several burrow entrances by the end of the 20 days (Appendix S12). This suggests that some species associations may have been overlooked, and longer monitoring durations may be necessary. Moreover, these results were obtained during the dry season and from burrows near roads. They therefore cannot be generalized to the entire park or to other seasons without caution. Finally, some significant preferential cohabitations may be false positives, given Fisher's exact test's 0.05 Type I error rate.

These results nonetheless highlight the remarkable extent of interspecific cohabitation in the current context of climate change. The involvement of antagonistic taxa suggests that climatic pressures could reinforce the value of burrows as refuges. Their benefits could therefore outweigh the risks associated with interspecific encounters. Moreover, given that the frequency and intensity of extreme maximum temperatures are increasing (Intergovernmental Panel on Climate Change 2023), it is plausible that their use will intensify and that the spectrum of species using them will expand. Preserving burrows, therefore, emerges as essential for maintaining biodiversity in the face of climatic variability.

### 4.2 Influence of Burrow Characteristics on Interspecific Cohabitation

Numerous factors influence burrows' capacity to buffer external climatic conditions, including their dimension, structural complexity and surface vegetation cover (Kirmiz 1962; Finlayson et al. 2005; Whittington‐Jones et al. 2011). All three factors significantly influenced cohabitation rates in our study.

Cohabitation rates increased with average burrow diameter. This contrasts with previous studies: Bulova (2002) found narrower desert tortoise burrows to be cooler and more humid, and Whittington‐Jones et al. (2011) reported greater temperature fluctuations in wider aardvark burrows than in narrower burrows of other species. A possible explanation is that wider burrows are more accessible to species of varying body sizes, favoring interspecific cohabitation despite less buffered conditions.

Burrows with more entrances were expected to host more individuals, owing to lateral chambers and improved ventilation. Multiple entrances allow the establishment of directional airflow, enhancing oxygen renewal within the burrow (Vogel et al. 1973). This is particularly relevant in hot environments, where high temperatures can raise metabolic oxygen demand (Pörtner 2001), whereas burrows have lower oxygen and higher carbon dioxide levels than the surface (Chu et al. 2021). However, our results showed the opposite relationship. The internal structure of burrows, not studied here, may constrain interspecific cohabitation. Indeed, Mukherjee et al. (2017) demonstrated a negative correlation between chamber height and the co‐occurrence of pythons and bats in India. Internal constraints may therefore outweigh the potential benefits of greater burrow structural complexity for interspecific cohabitation.

In terms of habitat characteristics, cohabitation rates increased with woody cover. Aardvarks generally favor open habitats (Skinner and Chimimba 2005). They have excellent hearing (Shoshani et al. 1988) and respond rapidly to distant predator sounds (Melton 1976). Because sound travels more easily through less dense vegetation (Hardt and Benedict 2021), aardvarks may detect predators earlier and have more time to escape. However, greater vegetation cover may mitigate extreme climatic conditions, making burrows more suitable refuges for multiple species. In contrast, geomorphological conditions favorable to aardvarks favor interspecific cohabitation. Cohabitation rates were higher in valleys than on plateaus, while aardvarks are frequently observed in flood‐prone habitats despite the risk of flooding (Skinner and Chimimba 2005), probably because of loose soils favorable for burrow excavation.

Importantly, our observations generally support the assumption that the monitored structures were aardvark burrows. Aside from the aardvark, the only species observed excavating at burrow entrances were the warthog and the porcupine. The warthog cannot excavate its own burrows (White and Cameron 2009), highlighting its role as a secondary modifier. The porcupine, although capable of digging, typically uses and modifies pre‐existing cavities, particularly abandoned aardvark burrows (Bordon et al. 2024). Given the presence of aardvarks, which frequently abandon burrows (Taylor and Skinner 2003), it is unlikely that these other species were the primary excavators. However, one case stands out: the abandoned termite mound. While this structure hosted only species already observed in other burrows, except for the yellow‐crowned gonolek (*Laniarius barbarous*), no aardvark ever entered it. Nevertheless, several aardvark‐related events of interest were recorded. This cavity in the abandoned termite mound is the only structure for which the aardvark does not appear to be the likely excavator, even though aardvarks have claws strong enough to breach termite mounds and the opening closely matches the dimensions of their typical burrows.

### 4.3 Zoonotic Risks

#### 4.3.1 Zoonotic Potential of Observed Cohabitations

The significant preferential cohabitations observed involved several taxa known to carry pathogens with zoonotic potential, including bats (*Chiroptera*), rodents ( *Cricetomys gambianus* , *Heliosciurus gambianus* , *Hystrix cristata* , and the Mice complex), and a non‐human primate ( *Erythrocebus patas* ). Indeed, these three orders host 76% of all known zoonotic viruses (Johnson et al. 2020). Their involvement in non‐random cohabitations may create opportunities for pathogen transmission between species sharing burrows, particularly when associations involve two taxa known to host pathogens with zoonotic potential, such as the *Chiroptera ×* Mice complex.

Other cohabitations should be considered based on bacteriological analysis of fecal samples. *Treponema*, a genus within the family Spirochaetaceae, was detected in feces from warthogs, porcupines, and baboons. Identification was performed at the genus level only, and *Treponema* includes numerous non‐pathogenic species. However, this genus also includes *T. pallidum* , the causative agent of syphilis, which has previously been identified in baboons (Centurion‐Lara et al. 2006; Mediannikov et al. 2020). Co‐occurrences of these species in the same burrows raise the question of possible interspecific transmission, although finer taxonomic identification is needed. This is particularly relevant given the frequent proximity of baboons and warthogs to human settlements: baboons are known to bite humans (World Health Organization 2024), while warthogs often inhabit areas near farms.

More generally, among the eight bacterial families with zoonotic potential detected, six occurred across multiple host species. Their distribution nonetheless varied among families. Two families were detected in only a single host category. In contrast, Bacillaceae showed the widest distribution, occurring in four host categories. Peptostreptococcaceae showed an intermediate pattern. This family was found in several hosts but was particularly frequent in porcupines (4/4). These differences may reflect variation in bacterial ecology and host associations. However, given the taxonomic resolution of the bacterial data and the small number of fecal samples (*n* = 13), these patterns should be interpreted with caution.

#### 4.3.2 Zoonotic Potential of Ticks

Considering that burrow use between species is predominantly successive, indirect interactions deserve attention, particularly regarding parasite transmission. Ticks were detected in five of the ten sampled burrows, distributed across four different vegetation types. The absence of collected ticks in five burrows does not guarantee an actual absence. Both collection methods have limitations: the shovel technique only allowed access to a shallow entrance depth, and the hot water bottle technique only covered a restricted portion of the entrance surface. More targeted approaches, such as wall aspiration or CO_2_‐baited traps, would likely have increased detection rates and reduced methodological bias (Jori et al. 2013), but are more complicated to deploy in the field.

Among all bacterial families including zoonotic pathogens detected in tick samples, Francisellaceae is the only one for which ticks are recognized as vectors. This family was consistently associated with the genus *Francisella*, which could include *F. tularensis* , the causative agent of tularemia (Ellis et al. 2002). Although our taxonomic resolution does not confirm the presence of *F. tularensis* , its ecology is relevant to our findings. Rodents often serve as reservoirs, while ticks can transmit the bacterium between hosts. Humans can become infected through tick bites, direct contact with infected animals, or exposure to contaminated environments (World Organization for Animal Health 2025). The systematic detection of *Francisella* in ticks, combined with the presence of rodents in burrows near human‐frequented areas (notably tourist lodge), therefore deserves attention from a One Health perspective.

Ticks are not recognized as vectors for the other zoonotic families identified. Indeed, they may ingest blood from infected animals without the bacteria developing or migrating to the internal organs required for transmission to a new host (Kitsou et al. 2021). Despite this, their presence in the samples remains relevant. The identification of zoonotic bacterial families in ticks containing warthog DNA indicates that these warthogs were likely carriers of the bacteria. This is supported by the identification of Bacillaceae in both ticks containing warthog DNA and in fecal samples from warthogs. In samples where no host DNA was detected, but zoonotic families were nonetheless identified, the bacteria may have been present on the tick's body surface (Narasimhan and Fikrig 2015; Khoo et al. 2016), likely due to environmental contamination within the burrow. In both scenarios, the findings suggest that the studied burrows were visited by animals carrying bacteria of zoonotic potential.

Therefore, although burrows are essential for survival in arid environments, our results show they contain wild hosts, vectors, and pathogens, creating potential hotspots for microbial exchange. Despite the limited number of fecal samples collected and burrows examined for ticks, bacterial families with zoonotic potential and ticks as candidate vectors were detected, supporting the hypothesis that these structures may contribute to zoonotic risk. From a “One Health” perspective, conservation and prevention should not be considered in silos (Narrod et al. 2012): preserving these structures is vital but requires monitoring interspecific interactions and managing human‐wildlife contact (Figure 3).

**FIGURE 3:**
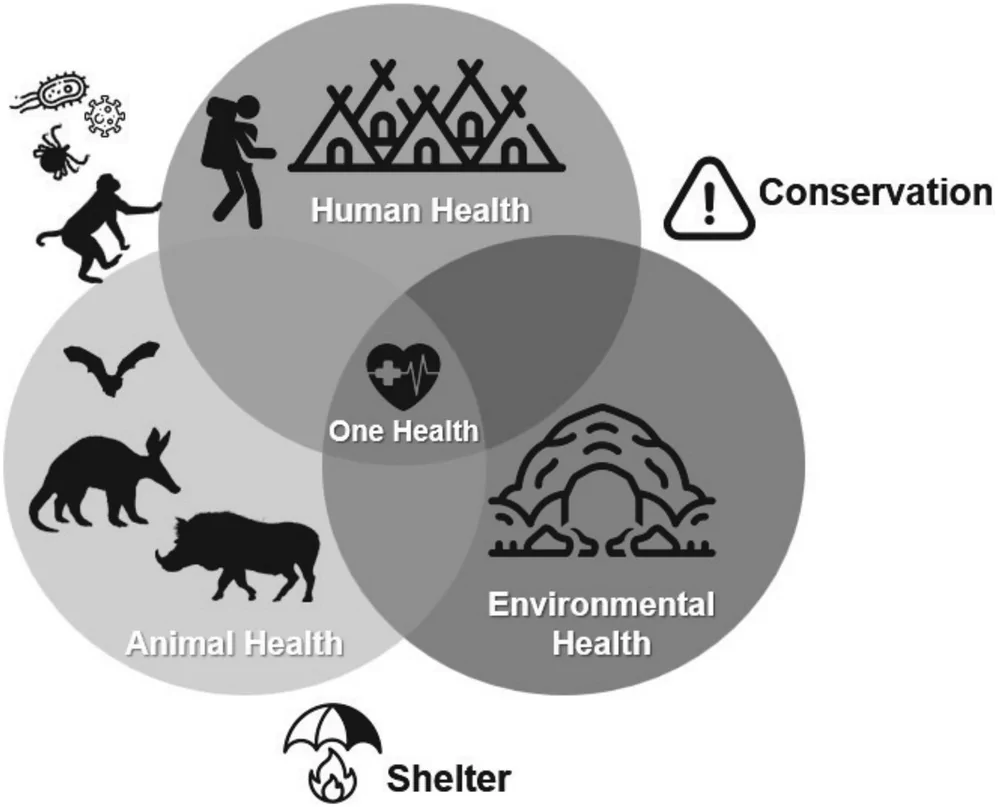
One Health framework applied to burrow ecosystems.

## 5 Conclusions and Perspectives

This study provides a comprehensive assessment of burrows use in Niokolo‐Koba National Park, based on 105 burrow entrances. Camera‐trap monitoring revealed the remarkable extent of interspecific burrow cohabitation. Thirty‐two species were recorded entering burrows, resulting in 172 species‐pair cohabitations. These ranged from shared ecological requirements to predator–prey associations, most of which occurred in temporal succession rather than simultaneously. However, the number of cohabitations varied among burrows, influenced by their environmental and structural characteristics. Based on these findings, burrow preservation should be recognized as a conservation priority, given their role as a critical shared resource for wildlife communities.

Regarding zoonotic implications, further research is needed to investigate the potential risks highlighted in this study. More extensive sampling and more precise identification of pathogens would help confirm these concerns. Nevertheless, the detection of *Treponema* in the feces of three species sharing the same burrows, combined with the systematic presence of *Francisella* in ticks, already supports the integration of sanitary surveillance into conservation strategies within a One Health framework.

## Author Contributions

**Clara Russo:** conceptualization (equal), data curation (equal), formal analysis (lead), investigation (equal), methodology (equal), software (lead), visualization (lead), writing – original draft (lead), writing – review and editing (equal). **Yves Brostaux:** formal analysis (supporting), methodology (equal), software (supporting), supervision (equal), validation (equal), writing – review and editing (equal). **Virginie Daelemans:** conceptualization (equal), data curation (equal), investigation (equal), methodology (equal), writing – review and editing (equal). **Simon Lhoest:** supervision (equal), validation (equal), writing – review and editing (equal). **Johan Michaux:** writing – review and editing (equal). **Mutien‐Marie Garigliany:** writing – review and editing (equal). **Julie Bonnald:** writing – review and editing (equal). **Fany Brotcorne:** writing – review and editing (equal). **Justine Broers:** writing – review and editing (equal). **Eric Heymans:** investigation (supporting), writing – review and editing (equal). **Philippe Taminiaux:** writing – review and editing (equal). **Ousmane Alioune Sylla:** writing – review and editing (equal). **Mallé Gueye:** writing – review and editing (equal). **Paul Moïse Diedhiou:** writing – review and editing (equal). **Cédric Vermeulen:** conceptualization (lead), funding acquisition (lead), methodology (equal), resources (lead), supervision (lead), validation (equal), writing – review and editing (equal).

## Funding

Funding was provided by C.R. (ULiège Mobility Fund, NiokoLodge/Ellëg) and V.D. (ARES‐CCD, NiokoLodge/Ellëg). The article publication charges were covered by C.V. using funds from the Laboratory of Tropical Forestry, University of Liège.

## Ethics Statement

Camera traps were installed in a manner that avoided any disturbance to wildlife, and sample collection was conducted only at the entrance of burrows, ensuring that no burrow was disturbed.

## Conflicts of Interest

The authors declare no conflicts of interest.

## Supporting information


**Appendix S1:** Textural analysis of burrow soil samples.
**Appendix S2:** Field data sheet for the characterization of burrows.
**Appendix S3:** Photographs illustrating the different categories of the burrow occupancy scale (A) Heavily frequented: smooted walls > 70% and fewer than 10 leaves (B) Moderately frequented: smooted walls < 50% and 10–20 leaves (C) Not frequented: smoothed walls < 50% and more than 20 leaves.
**Appendix S4:** Species accumulation curves for each burrow entrance monitored during the first study period (2023).
**Appendix S5:** Percentage of entrance (with ≥ 2 penetrating species) categorized by the number of penetrating species.
**Appendix S6:** Probability of observing at least one cohabitation as a function of filming duration where each point represents a single burrow entrance and the curve shows the fitted logistic regression.
**Appendix S7:** Summary table showing the number of independent detection events per species, categorized into three classes based on the level of interest: Class 1 (no interest), Class 2 (signs of interest), and Class 3 (entry into the burrow entrance).
**Appendix S8:** Characteristics of fecal samples.
**Appendix S9:** Presence‐absence matrix of bacterial families across fecal samples, families in bold represent those likely to contain zoonotic bacteria (− = not detected, + = detected).
**Appendix S10:** Characteristics of ticks collected at each entrance using two methods (1 = trowel, 2 = hot water bottle). Cells are shaded in gray for entrances where no ticks were found despite sampling.
**Appendix S11:** Presence‐absence matrix of bacterial families in tick samples from different burrow entrances (+ = detected in 1 sample, ++ = 2 samples). Families in bold represent those likely to contain zoonotic bacteria, and gray‐shaded columns indicate entrances where ticks contained *Phacochoerus africanus* DNA.
**Appendix S12:** Species accumulation curves for each burrow entrance monitored over 20 days during the second study period (2025).

## Data Availability

The raw database is not accessible online due to its considerable volume. However, it is not required for the demonstration presented in this article, as all cleaned data are available in the appendices. Exhaustive detections from camera traps are listed in Appendix S7, all bacterial families detected in biological samples are presented in Appendices S9 and S11.
