# Supplementary material for: Interspecific Cohabitation and Associated Zoonotic Risk in the Burrows of Niokolo‐Koba National Park, Senegal
Source: Ecol Evol. 2026 Oct 28;16(11):e74402. doi: 10.1002/ece3.74402 (PMC13717220; doi:10.1002/ece3.74402)
Supplement: Supplementary file 1 [file ECE3-16-e74402-s001.docx]

**Interspecific cohabitation and associated zoonotic risk in the burrows of Niokolo-Koba National Park, Senegal**

Clara Russo*^1^, Yves Brostaux^2^, Virginie Daelemans^1^, Simon Lhoest^1^, Johan Michaux^3,4^, Mutien-Marie Garigliany^5^, Julie Bonnald^6^, Fany Brotcorne^7^, Justine Broers^1^, Eric Heymans^8^, Philippe Taminiaux^8^, Ousmane Alioune Sylla^8^, Mallé Gueye^9^, Paul Moïse Diedhiou^10^, Cédric Vermeulen*^1^

^1^Forest is Life, Gembloux Agro-Bio Tech, University of Liège, Gembloux, Belgium ^2^Applied Statistics, Computer Science and Modeling Laboratory, Gembloux Agro-Bio Tech, University of Liège, Gembloux, Belgium ^3^Conservation Genetics Laboratory, University of Liège, Liège, Belgium ^4^University of Montpellier, CIRAD, INRAE, UMR 117 Animal Health, Territory, Risk and Environment, Montpellier, France ^5^Laboratory of Veterinary Pathology, FARAH - CIRM - INDEEP, University of Liège, Liège, Belgium ^6^Paris Zoological Park, National Museum of Natural History, Paris, France ^7^SPHERES Research Unit, Behaviour Biology Unit, Department of Biology, Ecology, and Evolution, Faculty of Sciences, University of Liège, Liège, Belgium ^8^Nioko Lodge, Tambacounda, Senegal ^9^Department of HydroSciences and Environment, Iba Der Thiam University of Thiès, Thiès, Senegal ^10^National Parks Directorate, Niokolo-Koba National Park, Tambacounda, Senegal.

*Correspondence: [russo.cl@outlook.com](mailto:russo.cl@outlook.com); [cvermeulen@uliege.be](mailto:cvermeulen@uliege.be)

**Appendices**

**Appendix 1.** Textural analysis of burrow soil samples

Five soil samples were collected during the first campaign at the entrances of burrows exhibiting distinct colors and/or textures. Soil was collected in a dry state and stored in 25 ml tubes, kept at ambient temperature. Soil texture was analyzed using the Bouyoucos hydrometer method to determine clay content, and by weighing to quantify sand content, following pretreatment with H₂O₂ and HCl to remove cementing agents (Beretta et al. 2014).

The analysis identified five distinct soil types forming a gradient of increasing clay content, ranging from sandy soils (types 1 and 2), through intermediate textures (types 3 and 4), to clay-rich compositions (type 5). Silt content remained consistently low across all types. These soil types were positioned on the USDA soil texture triangle:

Position of the five soil samples, corresponding to different burrow soil types encountered, marked by crosses on the USDA textural triangle (USDA, 1951)

Each burrow entrance monitored with a camera trap was assigned to one of the five soil types based on its visual resemblance to the soils previously analyzed.

**Appendix 2.** Field data sheet for the characterization of burrows

**Appendix 3.** Photographs illustrating the different categories of the burrow occupancy scale (A) Heavily frequented: smooted walls > 70 % and fewer than 10 leaves (B) Moderately frequented: smooted walls < 50 % and 10-20 leaves (C) Not frequented: smoothed walls < 50 % and more than 20 leaves

C

B

A

**Appendix 4.** Species accumulation curves for each burrow entrance monitored during the first study period (2023)

**Appendix 5**. Percentage of entrance (with ≥2 penetrating species) categorized by the number of penetrating species

**Appendix 6.** Probability of observing at least one cohabitation as a function of filming duration where each point represents a single burrow entrance and the curve shows the fitted logistic regression

**Appendix 7.** Summary table showing the number of independent detection events per species, categorized into three classes based on the level of interest: class 1 (no interest), class 2 (signs of interest), and class 3 (entry into the burrow entrance)

| **Species** | **1** | **2** | **3** | **Total** |
| --- | --- | --- | --- | --- |
| *Amphibia* | 0 | 1 | 0 | 1 |
| *Bucorvus leadbeateri* | 1 | 0 | 0 | 1 |
| *Camaroptera brachyura* | 2 | 10 | 13 | 25 |
| *Canis adustus* | 0 | 1 | 0 | 1 |
| *Centropus senegalensis* | 2 | 1 | 0 | 3 |
| *Cephalophus rufilatus* | 23 | 3 | 0 | 26 |
| *Chlorocebus sabaeus* | 4 | 6 | 0 | 10 |
| *Chiroptera* | 60 | 99 | 655 | 814 |
| *Coracias abyssinicus* | 3 | 0 | 0 | 3 |
| *Cossypha albicapilla* | 3 | 1 | 1 | 5 |
| *Cricetomys gambianus* | 5 | 4 | 7 | 16 |
| *Crocuta crocuta* | 2 | 9 | 2 | 13 |
| *Dicrurus adsimilis* | 1 | 1 | 0 | 2 |
| *Eremomela pusilla* | 0 | 1 | 2 | 3 |
| *Euxerus erythropus* | 19 | 18 | 7 | 44 |
| *Erythrocebus patas* | 10 | 18 | 1 | 29 |
| *Felis serval* | 0 | 1 | 0 | 1 |
| *Francolinus bicalcaratus* | 7 | 3 | 3 | 13 |
| *Galago senegalensis* | 3 | 0 | 0 | 3 |
| *Genetta genetta* | 38 | 21 | 1 | 60 |
| *Heliosciurus gambianus* | 1 | 13 | 8 | 22 |
| *Herpestes ichneumon* | 14 | 18 | 31 | 63 |
| *Hippotragus equinus* | 18 | 1 | 0 | 19 |
| *Hystrix cristata* | 44 | 75 | 27 | 146 |
| *Ichneumia albicauda* | 24 | 21 | 6 | 51 |
| *Kaupifalco monogrammicus* | 0 | 1 | 0 | 1 |
| *Kobus ellipsiprymnus defassa* | 10 | 7 | 0 | 17 |
| *Lagonosticta larvata* | 1 | 0 | 1 | 2 |
| *Lamprotornis chalybaeus* | 4 | 0 | 0 | 4 |
| *Laniarus barbarus* | 2 | 4 | 6 | 12 |
| *Lepus capensis* | 5 | 4 | 0 | 9 |
| *Malaconotus blanchoti* | 0 | 1 | 3 | 4 |
| *Mellivora capensis* | 4 | 20 | 10 | 34 |
| *Merops pusillus* | 0 | 0 | 7 | 7 |
| *Merops viridissinus* | 2 | 4 | 0 | 6 |
| *Mungos gambianus* | 0 | 1 | 6 | 7 |
| *Mungos mungo* | 9 | 18 | 12 | 39 |
| Mice complex | 16 | 24 | 52 | 92 |
| *Numida meleagris* | 80 | 98 | 24 | 202 |
| *Burhinus senegalensis* | 1 | 0 | 0 | 1 |
| *Orycteropus afer* | 28 | 77 | 110 | 215 |
| *Ourebia ourebi* | 3 | 4 | 0 | 7 |
| *Panthera leo* | 1 | 0 | 0 | 1 |
| *Panthera pardus* | 0 | 2 | 0 | 2 |
| *Papio papio* | 95 | 14 | 9 | 118 |
| *Phacochoerus africanus* | 34 | 80 | 157 | 271 |
| *Ptilopachus petrosus* | 18 | 55 | 58 | 131 |
| *Python sebae* | 0 | 0 | 1 | 1 |
| Lizards complex | 0 | 6 | 41 | 47 |
| *Sylvicarpa grimmia* | 0 | 1 | 0 | 1 |
| *Tchagra senegalus* | 0 | 3 | 2 | 5 |
| *Tockus erythrorhynchus* | 2 | 0 | 0 | 2 |
| *Tourterelle sp* | 3 | 0 | 0 | 3 |
| *Tragelaphus scriptus* | 55 | 20 | 0 | 75 |
| *Turdoides plebejus* | 1 | 1 | 0 | 2 |
| *Turdoides reinwardtii* | 3 | 7 | 2 | 12 |
| *Turtur abyssinicus* | 15 | 7 | 0 | 22 |
| *Uraeginthus bengalus* | 1 | 1 | 0 | 2 |
| *Varanus niloticus* | 0 | 1 | 0 | 1 |
| *Viverra civetta* | 0 | 2 | 5 | 7 |
|  |  |  |  |  |

**Appendix 8.** Characteristics of fecal samples

| **Feces ID** | **Collection date** | **Presumed species** | **Decomposition state** | **Distance to entrance** |
| --- | --- | --- | --- | --- |
| F1 | 06-03-23 | *Phacochoerus africanus* | Fresh, 1 day old | 5 m from g_30 |
| F2 | 08-03-23 | *Hystrix cristata* | Fresh, 2 days old | 10 m from g_10 |
| F3 | 21-03-23 | ? | Fresh, 1 day old | 0 m from g_10 |
| F4 | 21-03-23 | ? | Fresh, 1 day old | 0 m from g_10 |
| F5 | 02-04-23 | *Phacochoerus africanus* | Fresh, 1 day old | 25 m from g_38 |
| F6 | 02-04-23 | *Phacochoerus africanus* | Fresh, 1 day old | 25 m from g_38 |
| F7 | 02-04-23 | ? | Fresh, 1 day old | 10 m from g_48 |
| F8 | 02-04-23 | ? | Fresh, 1 day old | 1 m from g_39 |
| F9 | 05-02-25 | *Hippotragus equinus* | Fresh, 1 day old | 1 m from g_69 |
| F10 | 12-02-25 | *Phacochoerus africanus* | Fresh, 2 days old | 5 m from g_67 |
| F11 | 05-03-25 | *Hystrix cristata* | Fresh, 1 day old | 1 m from g_66 |
| F12 | 15-03-25 | *Papio Papio* | Fresh, 2 days old | 2 m from g_65 |
| F13 | 16-03-25 | *Hystrix cristata* | Fresh, 1 day old | 1m from g_62 |

**Appendix 9.** Presence-absence matrix of bacterial families across fecal samples, families in bold represent those likely to contain zoonotic bacteria (- = not detected, + = detected)

| **Bacterial families** | **F1** | **F2** | **F3** | **F4** | **F5** | **F6** | **F7** | **F8** | **F9** | **F10** | **F11** | **F12** | **F13** | **Total** |
| --- | --- | --- | --- | --- | --- | --- | --- | --- | --- | --- | --- | --- | --- | --- |
| *Acidaminococcaceae* | + | + |  | + | + |  |  | + |  |  |  |  |  | 5 |
| *Akkermansiaceae* |  | + |  |  |  |  | + | + |  |  |  |  |  | 3 |
| *Anaeroplasmataceae* | + |  |  |  | + | + |  |  | + |  |  |  |  | 4 |
| ***Bacillaceae 1*** | **+** |  | **+** |  |  |  |  | **+** | **+** | **+** | **+** |  | **+** | **7** |
| *Bacteroidaceae* | + | + | + | + | + | + | + | + | + |  |  |  |  | 9 |
| ***Campylobacteraceae*** |  |  | **+** | **+** |  |  |  |  |  |  |  |  |  | **2** |
| *Chitinophagaceae* | + |  |  |  |  | + |  |  |  |  |  |  |  | 2 |
| ***Clostridiaceae 1*** | **+** | **+** | **+** | **+** |  |  |  |  |  |  | **+** |  |  | **5** |
| *Desulfovibrionaceae* |  |  |  | + |  |  | + | + |  |  |  |  |  | 3 |
| *Eggerthellaceae* | + | + |  |  |  |  | + | + |  |  |  |  |  | 4 |
| ***Enterobacteriaceae*** |  |  | **+** | **+** |  |  | **+** |  |  |  |  |  | **+** | **4** |
| *Enterococcaceae* |  |  | + | + |  |  |  |  |  |  |  |  |  | 2 |
| *Erysipelatoclostridiaceae* |  |  |  | + |  |  |  |  |  |  |  |  |  | 1 |
| *Erysipelotrichaceae* | + |  |  | + | + | + | + | + |  |  | + | + |  | 8 |
| *Fibrobacteriaceae* | + |  |  |  |  | + |  |  |  |  |  |  |  | 2 |
| *Fusobacteriaceae* |  |  | + | + |  |  |  |  |  |  |  |  |  | 2 |
| *Lachnospiraceae* | + | + | + | + | + | + | + | + | + |  | + | + |  | 11 |
| *Lactobacillaceae* | + |  |  |  |  |  |  |  |  |  |  |  |  | 1 |
| *Metamycoplasmataceae* |  |  | + | + |  |  |  |  |  |  |  |  |  | 2 |
| *Methanobacteriaceae* |  | + |  |  |  |  |  |  |  |  |  |  |  | 1 |
| *Metanomassiliicoccaceae* |  |  |  |  |  |  |  | + |  |  |  |  |  | 1 |
| *Morganellaceae* |  |  | + |  |  |  |  |  |  |  |  |  |  | 1 |
| *Muribaculaceae* |  | + |  |  |  |  | + |  |  |  | + |  |  | 3 |
| ***Mycoplasmoidaceae*** |  | **+** |  |  |  |  |  |  |  |  |  |  |  | **1** |
| *Odoribacteraceae* |  |  |  | + |  |  | + | + |  |  |  |  |  | 3 |
| *Oligosphaeraceae* | + |  |  |  | + | + |  |  |  |  |  |  |  | 3 |
| ***Peptostreptococcaceae*** | **+** | **+** | **+** | **+** | **+** | **+** |  | **+** |  |  | **+** |  |  | **8** |
| *Planococcaceae* | + | + |  |  |  |  |  |  | + | + | + |  | + | 6 |
| *Porphyromonadaceae* | + | + |  | + |  |  | + | + |  |  |  |  |  | 5 |
| *Prevotellaceae* | + | + |  |  | + | + |  |  | + |  |  | + |  | 6 |
| *Rikenellaceae* |  | + |  | + |  |  | + | + | + |  |  |  |  | 4 |
| *Ruminococcaceae* | + | + | + | + | + | + | + | + | + |  | + | + |  | 11 |
| *Sphaerochaetaceae* | + |  |  |  | + | + |  |  |  |  |  |  |  | 3 |
| ***Spirochaetaceae*** | **+** | **+** |  |  | **+** | **+** |  |  |  |  |  | **+** |  | **5** |
| ***Streptococcaceae*** |  | **+** | **+** | **+** |  |  |  |  |  |  |  |  |  | **3** |
| *Succinivibrionaceae* |  | + |  |  |  |  |  |  |  |  |  |  |  | 1 |
| *Sutterellaceae* |  |  |  | + |  |  |  |  | + |  |  |  |  | 2 |
| *Victivallaceae* |  |  |  |  |  |  |  | + | + |  |  |  |  | 2 |
| *Clostridiales_Incertae Sedis XIII* |  |  |  |  |  |  |  |  | + |  |  |  |  | 1 |
| *Selenomonadaceae* |  |  |  |  |  |  |  |  |  |  |  | + |  | 1 |
| *Veillonellaceae* |  |  |  |  |  |  |  |  |  |  |  | + |  | 1 |
| *Paenibacillaceae 1* |  |  |  |  |  |  |  |  |  |  | + |  |  | 1 |

**Appendix 10.** Characteristics of ticks collected at each entrance using two methods (1 = trowel, 2 = hot water bottle). Cells are shaded in grey for entrances where no ticks were found despite sampling

| **Entrance ID** | **Date** | **Habitat** | **Ticks present** | **Method** | **Ticks Tube ID** | **Tick count** | |
| --- | --- | --- | --- | --- | --- | --- | --- |
| g_66 | 02-05-25 | Open forest | X | 1 | TK1 | 18 | |
|  |  |  |  | 2 | \ | \ | |
| g_67 | 02-05-25 | Savanna woodland | X | 1 | TK2 | 5 | |
|  |  |  |  | 2 | \ | \ | |
| g_69 | 02-27-25 | Savanna woodland | X | 1 | TK3 | 2 | |
|  |  |  |  |  | TK4 | 3 | |
|  |  |  |  |  | TK5 | 2 | |
|  |  |  |  |  | TK6 | 2 | |
|  |  |  |  | 2 | TK7 | 2 | |
|  |  |  |  |  | TK8 | 2 | |
| g_77 | 02-05-25 | Woody savanna |  |  |  |  | |
| g_80 | 02-27-25 | Bamboo stand | X | 1 | TK9 | 2 | |
|  |  |  |  | 2 | TK10 | 7 | |
| g_85 | 02-27-25 | Woody savanna | X | 1 | TK11 | 16 | |
|  |  |  |  |  | TK12 | 14 | |
|  |  |  |  |  | TK13 | 16 | |
|  |  |  |  | 2 | TK14 | 10 | |
| g_94 | 03-05-25 | Bamboo stand |  |  |  |  | |
| g_102 | 03-17-25 | Shrub  savanna |  |  |  |  | |
| g_103 | 02-27-25 | Shrub savanna |  |  |  |  | |
| g_105 | 03-26-25 | Open forest |  |  |  |  | |
| **TOTAL** |  | | | | | | **101** |

**Appendix 11.** Presence-absence matrix of bacterial families in tick samples from different burrow entrances (+ = detected in 1 sample, ++ = 2 samples). Families in bold represent those likely to contain zoonotic bacteria, and grey-shaded columns indicate entrances where ticks contained Phacochoerus africanus DNA

| **Bacterial families** | **g_69**  **(TK3-TK4)** | **g_80**  **(TK9-TK10)** | **g_85**  **(TK13)** | **g_66**  **(TK1)** | **g_67**  **(TK2)** | **Total** |
| --- | --- | --- | --- | --- | --- | --- |
| Atopobiaceae |  |  | + |  |  | 1 |
| **Bacillaceae** | **+** |  |  |  | **+** | **2** |
| Bifidobacteriaceae |  |  | + | + |  | 2 |
| **Corynebacteriaceae** |  |  | **+** | **+** | **+** | **3** |
| Dermacoccaceae | ++ |  |  |  |  | 2 |
| **Francisellaceae** | **++** | **++** | **+** | **+** | **+** | **7** |
| Gordoniaceae |  |  | + |  |  | 1 |
| Lawsonellaceae |  |  | + |  |  | 1 |
| Microbacteriaceae |  |  |  |  | + | 2 |
| **Moraxellaceae** | **+** |  |  | **+** | **+** | **2** |
| **Mycobacteriaceae** |  |  |  | **+** |  | **1** |
| Peptoniphilaceae |  |  | + |  |  | 1 |
| Prevotellaceae |  |  | + |  |  | 1 |
| Propionibacteriaceae |  |  | + | + |  | 2 |
| Pseudonocardiaceae |  |  |  | + |  | 1 |
| Sporichthyaceae |  |  |  | + |  | 1 |
| **Staphylococcaceae** | **+** |  | **+** | **+** |  | **3** |
| Weeksellaceae |  |  | + | + | + | 3 |

**Online resource 12.** Species accumulation curves for each burrow entrance monitored over 20 days during the second study period (2025)

**References**

Beretta, A. 2014. “Soil Texture Analyses Using a Hydrometer: Modification of the Bouyoucos Method.” International Journal of Agriculture and Natural Resources 41: 263–271.
